# Amazon climatic factors driving terpene composition of *Iryanthera polyneura* Ducke in *terra-firme* forest: A statistical approach

**DOI:** 10.1371/journal.pone.0224406

**Published:** 2019-11-12

**Authors:** Erika Ramos Martins, Ingrit Elida Collantes Díaz, Mateus Luís Barradas Paciencia, Sergio Alexandre Frana, Márcia Ortiz Mayo Marques, Damila Rodrigues de Morais, Marcos Nogueira Eberlin, Ivana Barbosa Suffredini

**Affiliations:** 1 Graduate Program in Environmental and Experimental Pathology, Paulista University, São Paulo, São Paulo, Brazil; 2 Chemistry Engineering Department, Chemistry and Textile Engineer Faculty, Engineer National University, Lima, Peru; 3 Center for Research in Biodiversity, Paulista University, São Paulo, São Paulo, Brazil; 4 Agronomic Institute, São Paulo State, Campinas, São Paulo, Brazil; 5 ThomSon Laboratory, Chemistry Institute, Universidade de Campinas – UNICAMP, Campinas, São Paulo, Brazil; 6 School of Engineering, Mackenzie Presbyterian University, São Paulo, São Paulo, Brazil; Higher Institute of Applied Sciences and Technology of Gabes University of Gabes, TUNISIA

## Abstract

*Iryanthera polyneura* (Myristicaceae) is popularly known as *cumala-colorada*, and can be found in the Amazon rain forest. The present study aimed the evaluation of the chemical composition of the essential oils (EOs) obtained from the leaves of *I*. *polyneura* throughout a two-year period in order to correlate chemical findings with seasonality and climatic variation. Leaves from *I*. *polyneura* were collected 15 times from three different individuals, identified as 22EO, 80EO and 530EO, between the years of 2009 and 2011. The EOs were obtained and tested by gas-chromatography-mass spectrometry techniques. ANOVA and multivariate analyses were used to assess the relationship between the percentage of terpenes and seasonal/climatic variations. Fifty-nine compounds were detected in the EOs, of which 44 were identified (74.5%). The main components of the EOs were spathulenol (6.42 ± 1.02%), α-cadinol (5.82 ± 0.40%) and τ-muurolol (5.24 ± 0.03%). Higher levels of spathulenol were observed in 22EO during rainy season, while τ-muurolol occurred in high amounts during the dry season, as did α-cadinol in 22EO and 80EO. Correspondence analysis revealed a distinction between two groups of EOs based on seasonality, whereas a canonic correspondence analysis and ANOVA determined how the major compounds were related to both seasonality and climatic factors. Non-metric multidimensional scaling in association with an analysis of similarities showed that the abundance and composition of terpenes in the samples varied moderately among the three *Iryanthera* individual plants. Present findings have shown that there is variation in the occurrence of the major compounds spathulenol, τ-muurolol and α-cadinol produced by *I*. *polyneura* and that the pattern of variation is related to season and climatic changes.

## Introduction

Brazil is the richest country in terms of biodiversity in the world. Currently, the Brazilian flora harbors at least 46,500 species, of which 4,754 are algae, 33,158 are angiosperms, 1,567 bryophytes, 5,719 fungi, 30 gymnosperms and 1,345 are ferns and lycophytes [1]. As living organisms, plants communicate with the environment, and some of their strategies are related to defense [2] and others to reproduction [3].

EOs are an organic mixture of compounds occurring in specialized plant cells or glands, that play a fundamental role in plant-biotic/abiotic environment interactions [4]. They originate from intermediate products of the main metabolic pathways involved in the production of sugars, lipids and proteins. Characteristically, EOs have high genetic plasticity and wide chemical diversity, which allows the plants to adapt to the environmental demands, which are in continuously changing [5]. Among the functions exerted by EOs in plants, defense against bacteria, virus and fungi, as well as herbivorous insects or animals have been well-described [6].

Plants within the Myristicaceae have been known to produce EOs. Ethnopharmacological interest in this family emerged in the 1950’s, when Richard Schultes disclosed the discovery that some species of the genus *Virola* were sources of snuffs *yakee*, *parika* or *epena*, a powerful hallucinogenic narcotic used by Indian people from the Amazon region [7]. Myristicaceae species are still used for many purposes by native Amazonians, for example, in the preparation of arrows and for the treatment of dermatological or stomach infections, rheumatism, tumors, among others [8, 9, 10]. Some species of the genus *Iryanthera* have been popularly used as hallucinogenic, such as the bark of *I*. *macrophylla* (Benth.) Warb., or used as antidiarrhoeic, as the bark of *I*. *tessmanii* Markgr.; moreover, the wood of several species has been used in construction [11]. A phytochemical study made with the fruits of *I*. *lancifolia* Ducke revealed the presence of dihydrochalcones and flavonolignans which showed higher antioxidant activity in relation to α-tocopherol and quercetin [12]. *I*. *polyneura* were chemically accessed [13], and diarylpropanoids were isolated from the wood of their trunks.

Due to the lack of chemical and biological information regarding *I*. *polyneura*, the aim of the present work was to assess the chemical composition of the EOs obtained from the leaves of *I*. *polyneura* throughout a two-year span. Further, researchers aimed at the comprehension of how climatic factors such as the relative humidity, precipitation, minimum, average and maximum daily temperatures, insolation, wind and evaporation, and the seasonal variation might affect the chemical composition of the oils.

## Material and methods

### Collection of botanical material

The collection of botanical material was carried out in a *terra-firme* forest, within the Amazon Basin. The collections were made under licenses # 14895 (ICMBio/MMA/Brazil) and 12A/2008 (IBAMA/CGen/MMA), as required by the Brazilian Government. A voucher from each individual was collected as described in Table 1. The present study was based on a previous one made with *Osteophloeum platyspermum* (Spruce ex A.DC.) Warb., Myristicaceae [14].

**Table 1:** Indexation of botanical material collected from *Iryanthera polyneura*. DBH = diameter at breast height.

Individual (Field Number)	Herbarium Voucher	Determined by	DBH (cm)	Universal Transverse Marcator (UTM Zone 20)	
				Longitude (X)	Latitude (Y)
22	Oliveira A.A. 4064; UNIP 5170	Paciencia, M.	14.1	780103.21	9686375.79
80	Oliveira 4144; UNIP 5279	Paciencia, M.	17.4	780073.14	9686558.12
530	Paciencia, M. 3609; UNIP 8566	Paciencia, M.	14.5	780141.24	9686608.38

Collections were made from trees having DBH≥10cm (diameter at breast height), closely located in the same hectare plot, containing enough leaf biomass to allow for a periodical collection spanning two years, from October 2009 to December 2011. The individuals were identified as 22EO, 80EO and 530EO. Individuals 22EO and 530EO provided 15 material collections, while 80EO provided 14. The collection dates were determined in a random way, depending on the expedition logistics.

Samples of the collected material for botanical research were deposited at the UNIP Herbarium (Table 1), São Paulo, Brazil. The material was cleaned so as to remove contaminants such as insects or other animals, other organs of the same plant, other plants, sand and earth. The cleaned material was kept in a cold room until it was used to obtain the volatile oils.

### Preparation of the EOs

Fresh leaves of *I*. *polyneura* were periodically collected from the same individuals in order to obtain EOs. EOs were obtained by hydrodistillation in a Clevenger apparatus [15] for four hours. The oils were collected from the Clevenger apparatus with the aid of pentane, and were evaporated using a rotary evaporator apparatuses (Buchi, Switzerland). The remaining water was then removed with the addition of anhydrous sodium sulfate. The yields (mg/mg) of all EO samples (15 samples obtained from individuals 22EO, and, 14 samples obtained from individual 80EO and 530EO) were evaluated and then stored at -10°C, until use. To perform gas chromatography coupled to mass spectrometer analyses (GC-MS), 20μL of each oil were diluted in 980μL of acetone.

### Analysis of volatile oils by gas chromatography coupled to mass spectrometry (GC-MS)

GC-MS analyses were performed on a Shimadzu 14B/QP5050A apparatus, with quadrupole type analyzer of the same brand. The column used was BPX5 (non-polar 5% Phenyl Polisylphenlene Siloxane), 30m, with internal diameter of 0.25mm. The running conditions of the gas chromatography were: initial oven temperature 60°C (6 min), final temperature 320°C (8 min), temperature increase of 10°C/min and a total running time of 40 min, column pressure was 150.0kPa with a column flow of 2.5mL/min, linear velocity of 58, split ratio of 9 and total flow of 30.0mL/min. Each of the 44 acetone-diluted samples were injected at a volume of 1μL. The substances were identified by comparing the obtained mass spectra with libraries, such as Willey229, NIST107, SHIM1607 and NIST21, and with a reference books and retention indices [16].

The retention indices (RI) were obtained from the injection of a mixture of n-alcanes (C_9_H_20_–C_25_H_52_, Sigma Aldrich, 99%) using Van den Dool and Kratz equation [17].

### Experimental design

With three exceptions, yields (mg/mg) of all EO samples were obtained. Fifteen samples were obtained from individuals 22EO and 530EO and 14 samples obtained from individual 80EO.

The chemical and statistical variation were calculated from 41 samples of EOs (15 from 22EO, 12 from 80EO and 14 from 530EO) and were based on terpene percentages. Two EOs from individual 80EO and one EO from individual 530EO were discarded because the number of terpenes detected after GC-MS analysis was very low. Therefore, the samples were considered outliers, since they created trends in the analyses in a very significant way. For the multivariate analyses, 41 EOs were considered as the cases and the 59 terpenes as the variables. Climatic variables used for running the analyses were relative humidity, precipitation, daily temperatures (minimum, average and maximum), insolation, wind speed and evaporation, where data obtained from the day of collection was considered (www.inpe.gov.br). Seasons in the Amazon rain forest are defined according to the rain intensity, so the period of the year when the rain is more intense (accumulated precipitation as high as 3,500mm/year) is defined as the rainy season (RS), popularly known as the “winter”, and spans from January to July. The period of the year known as “summer”, or dry season (DS), is a period in which the precipitation drops down to 900mm/year and spans from July to December [18, 19].

### Statistical analyses

One-way ANOVAs and t-tests were performed in order to compare the occurrence of the terpene levels among individuals and between seasons. Differences were considered significant if α<0.05 [20]. NMDS analysis was performed in the association with ANOSIM test of hypotheses, which is similar to the non-parametric analysis of variance based on similarities among samples [21]. In the present case we used ANOSIM to test two different null hypotheses (H_0_). The first was that there were no differences in the percentual-abundance and the composition of terpenes of the three *Iryanthera* individual plants (22EO, 80EO, and 530EO). The second was that there was no difference between EO samples obtained during dry and rainy seasons. Correspondence analysis (CA) and canonical correspondence analysis (CCA) were also conducted using the software MVSP, Multivariate Statistical Package version 3.22 (Kovach Computing Services) and Primer 6 version 6.1.6 (Primer-E Ltd.).

## Results and discussion

**Fig 1:**
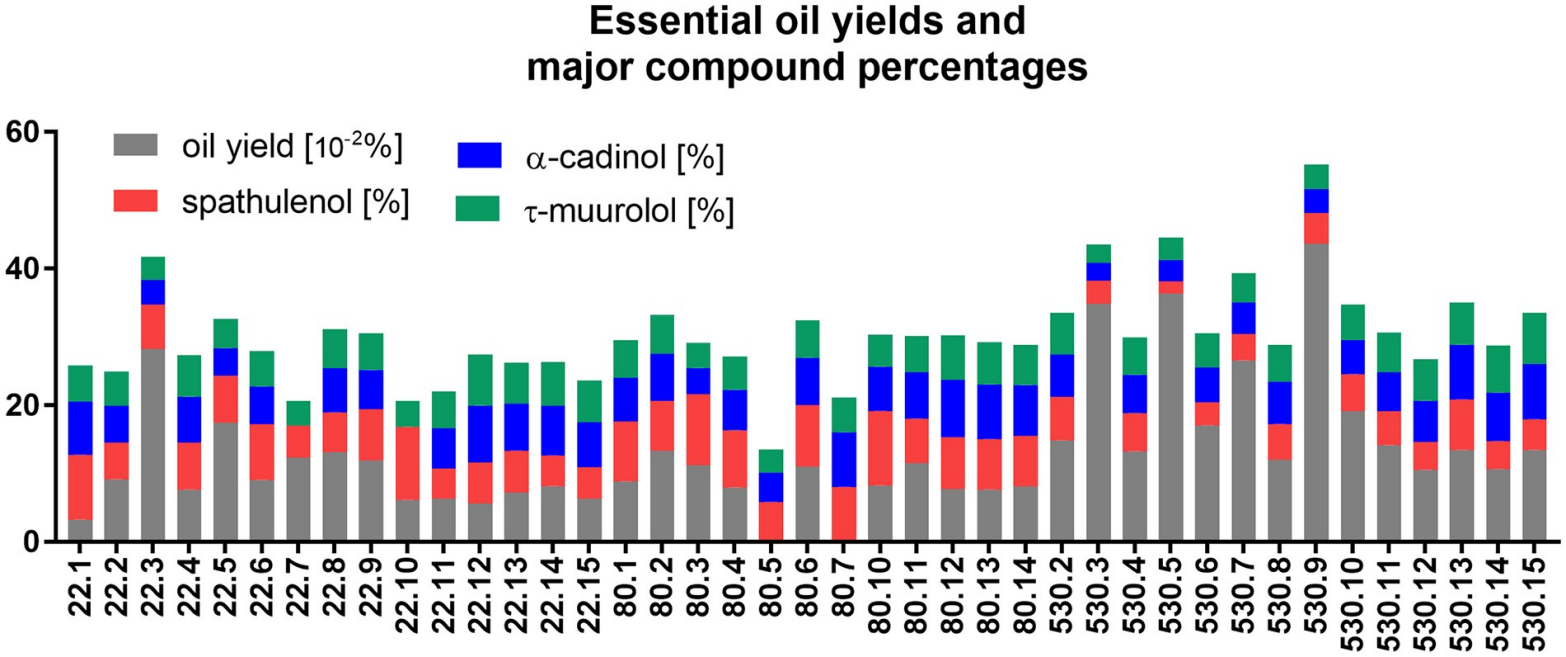
Yields from the essential oils obtained from the *Iryanthera polyneura* leaves and the percentage of the major compounds spathulenol, α-cadinol and τ-muurolol present.

Yields of the volatile oils obtained from the leaves of three individuals of *Iryanthera polyneura* spanned from 0.03% to 0.43%, with the lowest yield observed for 22EO1 and the highest for the 530EO9 (Table 2, Fig 1). The yields of the oils from individuals 80EO and 530EO were significantly higher than the oils from the individual 22EO (*F*_(2,39)_ = 5.238; *P* = 0.0097).

**Table 2:** Yields (%) obtained for the essential oils obtained from the leaves of three different *Iryanthera polyneura* individual plants. #22, #80 and #530 identifies different individuals, while EO1 toEO15 identifies dates of collection. Essential oil yield means for each individual was compared using one-way ANOVA, followed by Tukey post-test, considered significant if α<0.05.

Essential oil ID	Yield (%)	Essential oil ID	Yield (%)	Essential oil ID	Yield (%)	Date of collection
22EO1	0.033	80EO1	0.0889	530EO1	0.1041	10/01/2009
22EO2	0.0916	80EO2	0.1332	530EO2	0.1487	11/01/2009
22EO3	0.2821	80EO3	0.1124	530EO3	0.3483	02/03/2010
22EO4	0.0769	80EO4	0.0799	530EO4	0.1325	03/12/2010
22EO5	0.1745	80EO5	NT	530EO5	0.3635	05/15/2010
22EO6	0.0907	80EO6	0.1104	530EO6	0.1708	05/28/2010
22EO7	0.1232	80EO7	NT	530EO7	0.2657	08/29/2010
22EO8	0.1313	80EO8	0.0767	530EO8	0.1201	11/05/2010
22EO9	0.1195	80EO9	0.3378	530EO9	0.4362	12/14/2010
22EO10	0.0617	80EO10	0.0828	530EO10	0.191	02/11/2011
22EO11	0.0631	80EO11	0.115	530EO11	0.1419	04/15/2011
22EO12	0.0561	80EO12	0.0774	530EO12	0.1058	07/08/2011
22EO13	0.0723	80EO13	0.0767	530EO13	0.1346	08/20/2011
22EO14	0.0817	80EO14	0.0814	530EO14	0.107	10/21/2011
22EO15	0.0633			530EO15	0.1348	12/16/2011
mean±SD	0.10±0,02^A^	mean±SD	0.11±0,02 ^A^	mean±SD	0.19±0,03^B^	
**Essential oil yield comparison**						
Barlett’s statistic corrected		4.479	*P* = 0.1065			
One-way ANOVA/Tukey		*F*_(2,39)_ = 5.238	***P* = 0.0097**			
Multiple comparisons		22EO vs. 80EO	*P* = 0.9154			
		22EO vs. 530EO	***P* = 0.0120**			
		80EO vs. 530EO	***P* = 0.0487**			

^A^ = no differences among means;

^B^ = differences among means. NT = not tested.

**Fig 2:**
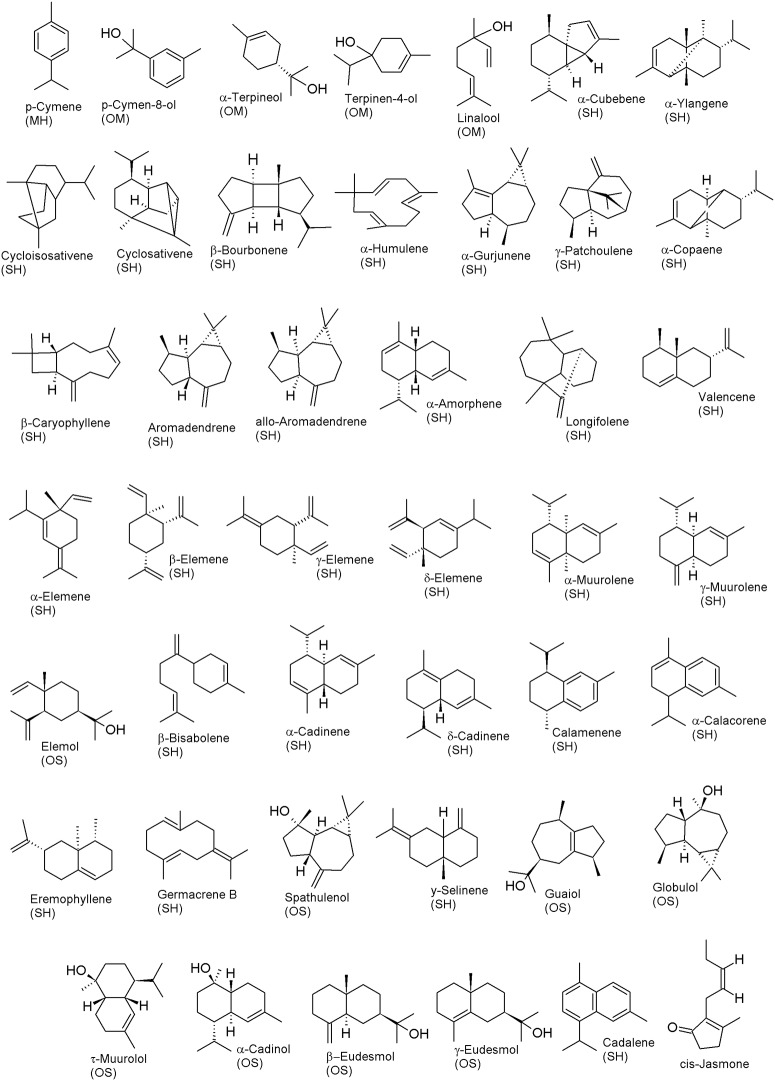
Structure of the compounds identified in the essential oils from the leaves of *Iryanthera polyneura*. MH = monoterpene hydrocarbon; OM = oxygenated monoterpene; SH = sesquiterpene hydrocarbon; OS = oxygenated sesquiterpene.

GC-MS analyses of the 44 EOs showed that terpenes were the only type of compound that was identified for this species in the present analysis (Table 3, Fig 2), unlike the possible occurrence of phenylpropanoids in some species of Myristicaceae, such as *Myristica fragrans* [22]. Sesquiterpene hydrocarbons prevailed in the oils of the three *I*. *polyneura* individual plants, followed by the presence of oxygenated sesquiterpenes. Forty four terpenes, or 74.5%, were identified, and 15 compounds were not determined (25.4%). Spathulenol (6.42 ± 1.02%), α-cadinol (5.82 ± 0.40%) and τ-muurolol (5.24 ± 0.03%) were the major compounds found in the EOs (*N* = 44). Suffredini et al. (2016) has already reported the highest percentages of spathulenol and α-cadinol in EOs from another species of Myristicaceae, such as *Osteophloeum platyspermum* Warb.

**Table 3:** Chemical composition (in percentage, %) of essential oils obtained from individual *I*. *polyneura* leaves from 22EO, 80EO and 530EO individuals and a summary of the class of compounds identified.

			22EO		80EO		530EO	
Compound	RI	RI ref.	Mean	Standard error	Mean	Standard error	Mean	Standard error
p-Cymene	1027	*	0.01	0.01	0.02	0.01	0.01	0.01
Linalool	1109	*	0.06	0.03	0.03	0.03		
Terpinen-4-ol	1182	*	0.03	0.03	0.02	0.02		
p-Cimen-8-ol	1190	*	0.02	0.01				
α-Terpineol	1195	*	0.06	0.02	0.03	0.02	0.01	0.01
δ-Elemene	1337	1335	1.08	0.17	0.97	0.10	1.44	0.22
α-Cubebene	1349	1345	0.10	0.03	0.21	0.15	0.17	0.04
Cycloisosativene	1364	*	0.35	0.10	0.45	0.09	0.52	0.09
Cyclosativene	1366	1369	0.98	0.26	0.46	0.10	1.20	0.25
α-Yllangene	1370	1373	0.12	0.04	0.06	0.03	0.24	0.06
α-Copaene	1375	1374	0.43	0.09	0.51	0.05	0.68	0.12
β-Bourbonene	1383	1387	0.63	0.14	0.33	0.06	0.37	0.07
β-Elemene	1391	1389	2.56	0.28	3.84	0.25	3.35	0.18
β-Caryophyllene	1417	1417	0.64	0.09	0.63	0.10	0.81	0.11
Sesq1	1428	*	0.72	0.13	1.26	0.20	0.98	0.19
γ-Elemene	1433	1434	0.31	0.05	0.50	0.08	0.59	0.08
Aromadendrene	1437	1439	0.65	0.08	1.36	0.11	1.03	0.10
α-Humulene	1452	1452	0.13	0.04	0.16	0.05	0.17	0.03
α-Gurjunene	1408	1409	0.01	0.01	0.06	0.03	0.08	0.03
α-Patchoulene	1454	1454	0.01	0.01			0.08	0.07
Allo-Aromadendrene	1459	1458	0.12	0.03	0.30	0.05	0.26	0.04
γ-Muurolene	1475	1478	2.85	0.28	2.90	0.15	3.90	0.33
α-Amorphene	1479	1483	1.25	0.15	1.28	0.12	1.85	0.23
Sesq2	1484	*	0.65	0.09	0.98	0.05	0.93	0.07
α-Elemene	1493	*	1.36	0.12	1.70	0.11	1.94	0.15
α-Muurolene	1499	1500	2.80	0.30	3.12	0.28	4.03	0.41
β-Bisabolene	1508	1505					0.01	0.01
α -Cadinene	1513	1513	1.53	0.32	1.73	0.07	2.10	0.23
Sesq3	1514	*	0.69	0.27	0.09	0.09	0.33	0.21
Calamenene	1522	1521	1.92	0.15	1.25	0.14	2.41	0.27
Valencene	1533	1532	0.61	0.07	0.85	0.06	1.00	0.07
α-Cadinene	1536	1537	0.10	0.06				
α-Calacorene	1541	1544	0.60	0.14	0.31	0.08	0.30	0.10
Elemol	1549	1548	2.08	0.24	1.13	0.16	1.11	0.12
Germacrene B	1555	1559	0.72	0.13	1.07	0.28	0.97	0.15
Longifolene	1558	1555	0.35	0.03	0.80	0.08	0.41	0.07
Sesq4	1565	*	3.00	0.22	4.15	0.34	4.17	0.32
Spathulenol	1577	1577	6.51	0.47	8.13	0.42	4.61	0.37
Sesq5	1582	*	4.79	0.21	7.14	0.40	5.09	0.43
γ-Selinene	1590	*	1.03	0.13	2.00	0.17	1.04	0.14
Guaiol	1597	1600	0.79	0.13	0.13	0.04	0.18	0.03
Globulol	1601	1590	1.30	0.15	2.62	0.21	0.74	0.24
Eremophyllene	1601	*	0.05	0.05			1.05	0.21
Sesq6	1611				0.78	0.11		
Sesq7	1624	*	1.87	0.26	1.89	0.15	1.91	0.22
Sesq8	1628	*	5.19	0.47	3.58	0.30	4.33	0.25
Sesq9	1631	*	1.82	0.26	1.29	0.06	1.37	0.12
τ-Muurololl	1641	*	5.28	0.29	5.19	0.26	5.26	0.37
Sesq10	1646	*	2.46	0.25	2.45	0.11	2.87	0.19
β-Eudesmol	1649	1649	1.28	0.22	0.89	0.04	0.76	0.05
α-Cadinol	1654	1654	5.35	0.65	6.61	0.41	5.49	0.45
γ-Eudesmol	1664	1630	0.07	0.04			0.16	0.06
Sesq11	1667	*	0.19	0.13	0.74	0.17	1.16	0.18
Cadalene	1673	1675	1.13	0.12	0.98	0.09	1.41	0.09
Sesq12	1693	*	0.15	0.06	0.81	0.10	0.86	0.11
Sesq13	1706	*	0.23	0.08	0.46	0.02	0.31	0.03
Sesq14	1716	*	0.67	0.16	0.88	0.15	0.70	0.16
cis-Jasmone	1725	*	0.01	0.01				
Sesq15	1748	*	0.09	0.05	0.30	0.03	0.24	0.03
Monoterpene hydrocarbon (%, MH)			0.69		0.85		0.47	
Oxigenated monoterpene (%, OM)			3.70		1.42		0.23	
Sesquiterpene hydrocarbon (%, SM)			78.24		80.74		81.82	
Oxigenated sesquiterpene (%, OS)			17.36		17.00		17.48	
*MH OM SH OS amounts*								
	MH (A)	0.67	0.1102	F (3,8) = 2580		**<0.0001**		
	OM (B)	1.783	1.018		A-B	0.7197		
	SH (C)	80.27	1.06		A-C	**<0.0001**		
	OS (D)	17.28	0.1442		A-D	**<0.0001**		
					B-C	**<0.0001**		
					B-D	**<0.0001**		
					C-D	**<0.0001**		

Sesq = unidentified sesquiterpenes;

* = not found

Table 3 shows the qualitative and quantitative terpene variation in individuals 22EO, 80EO and 530EO. Cis-jasmone and α-cadinene were solely found in 22EO, while sesq6 exclusively occurred in 80EO and β-bisabolene in 530EO. This difference may suggest that each individual has genetic variability or that each one may be in a particular physiological phase influenced by intrinsic or extrinsic factors. Spathulenol, elemol, α-muurolene, τ-muurolene, sesq2, sesq3, and τ-muurolol were commonly found in all individuals. Hydrogenated and oxygenated monoterpenes (MH and OM) and sesquiterpenes (SH and OS) were identified in the oils obtained from the three individuals. The terpene content in each of the four groups was summed and used in the statistical evaluation. According to the ANOVA, the SH-group was significantly more prevalent in the oils, in relation to the other three groups (*F*_(3,8)_ = 2.580; *P* < 0.001), as was the group OS.

Results obtained from the statistical analyses of the chemical composition percentage and seasonal variation were assessed. A *t*-test was performed in order to evaluate variation between terpene levels in relation to seasonality, and a F test to compare variances was applied to all *t*-tests (*P* > 0.05). For that analysis, the total terpene percentages were summed for each of the 41 oils, which produced two groups, those EOs from the DS and those from the RS. The analyses were made for each set of oils, 22EO, 80EO and 530EO. The total terpene percentage of oils occurring in 22EO was significantly lower in DS samples (*t*_0.05(2),12_ = 3.603; *P* = 0.0036), but higher for the oils from 80EO (*t*_0.05(2),9_ = 2.305; *P* = 0.0467), while no differences were observed in the terpenes from 530EO (*t*_0.05(2),12_ = 1.295; *P* = 0.2197). When considering the total amount of terpenes of all individuals, which were pooled together to evaluate a variation for the species, no differences were observed between the RS and DS (*t*_0.05(2),13_ = 0.6985; *P* = 0.4972).

Statistical analyses were also made considering only the terpenes that were common to all of the 41 EOs, τ-muurolene, α-muurolene, elemol, spathulenol, τ-muurolol, sesq 2 and sesq 3. The analysis revealed that the common terpenes occurred in higher amounts in the DS (*t*_0.05(2),37_ = 2.078; *P* = 0.0447).

Spathulenol, τ-muurolol and α-cadinol were the major compounds identified. As seen in Table 4, the occurrence of spathulenol was measured in each individual, according to the seasonality. It was observed that spathulenol was higher in the RS for individual 22EO (*t*_0.05(2),11_ = 2.345; *P* = 0.0388), but in individuals 80EO and 530EO, levels were not affected by the time of year of collection. The occurrence of spathulenol among the three individuals and the differences between the RS and DS were evaluated. In the analyses, the content of spathulenol was significantly higher in both the RS (*F*_(2,19)_ = 10.82; *P* = 0.0007) and the DS (*F*_(2,16)_ = 5.911; *P* = 0.012), in the oils derived from individual 80EO. The occurrence of τ-muurolol was also evaluated for each individual according to the seasonality, and it was observed that the occurrence of this terpene was significantly higher during DS, for 22EO (*t*_0.05(2),11_ = 2.311; *P* = 0.0412), 80EO (*t*_0.05(2),10_ = 3.012; *P* = 0.0131) and 530EO (*t*_0.05(2),11_ = 2.349; *P* = 0.0385). The total amount of τ-muurolol calculated for the three individuals together was seasonally compared, and a significantly higher level of production of the terpene during the DS vs the RS was confirmed (*t*_0.05(2),46_ = 2.22; *P* = 0.0314). Finally, the seasonal variation of α-cadinol differed from what was observed for spathulenol and for τ-muurolol. The percentage of α-cadinol was higher in the DS for individuals 22EO (*t*_0.05(2),11_ = 2.436; *P* = 0.0331) and 80EO (*t*_0.05(2),10_ = 2.912; *P* = 0.0155), while the compound was predominant in RS in individual 530EO (*t*_0.05(2),11_ = 2.257; *P* = 0.0453). The total amount of α-cadinol produced by all three individuals together, when seasonally compared, resulted in a significant higher occurrence of α-cadinol in DS (*t*_0.05(2),39_ = 2.674; *P* = 0.0109).

**Table 4:** Statistical analyses based on the chemical composition (in percentage, %) of the essential oils obtained from the leaves of individuals 22EO, 80EO and 530EO of *Iryanthera polyneura* in either the dry season (DS) or the rainy season (RS).

Analysis	factors	mean	SE	F / t	comparison	P/adjusted P	Analysis	factors	mean	SE	F / t	comparison	P/adjusted P	Analysis	factors	mean	SE	F / t	comparison	P/adjusted P
*Comparison of the percentage means of the major compounds occurring in the rainy and the dry seasons of each individual plant*																				
*spathulenol*							*α-cadinol*							*τ-muurolol*						
t-tests	22EO (A)			t = 2.345 df = 11		**0.0388**	t-tests	22EO (A)			t = 2.436 df = 11		**0.0331**	t-tests	22EO (A)			t = 2.311 df = 11		**0.0412**
	RS	7.307	0.6993					RS	5.466	0.4597					RS	4.793	0.3727			
	DS	5.428	0.2749					DS	7.012	0.4258					DS	5.993	0.3538			
	80EO (B)			t = 0.8917 df = 10		0.3935		80EO (B)			t = 2.912 df = 10		**0.0155**		80EO (B)			t = 3.012 df = 10		**0.0131**
	RS	8.51	0.8227					RS	5.695	0.5491					RS	4.593	0.3467			
	DS	7.748	0.2299					DS	7.533	0.3114					DS	5.79	0.194			
	530EO (C)			t = 1.314 df = 12		0.2135		530EO (C)			t = 2.257 df = 11		**0.0453**		530EO (C)			t = 2.349 df = 11		**0.0385**
	RS	4.199	0.4534					RS	4.83	0.6215					RS	4.439	0.46			
	DS	5.162	0.5943					DS	2.948	0.309					DS	5.833	0.3482			
*Comparison of the percentage means of major compounds occurring in the rainy season among the three individual plants*																				
*spathulenol*							*α-cadinol*							*τ-muurolol*						
ANOVA 1-factor				F (2,19) = 10.82		**0.0007**	ANOVA 1-factor				F (2,17) = 2.068		0.1571	ANOVA 1-factor				F (2,19) = 0.1554		0.8572
	22EO (A)	6.948	0.7043		A-B	0.2564		22EO (A)	5.466	0.4597		A-B	0.9444		22EO (A)	4.959	0.3629		A-B	0.8446
	80EO (B)	8.51	0.8227		A-C	**0.0154**		80EO (B)	5.695	0.5491		A-C	0.2702		80EO (B)	4.593	0.3467		A-C	0.9728
	530EO (C)	4.199	0.4534		B-C	**0.0007**		530EO (C)	4.37	0.4825		B-C	0.179		530EO (C)	4.823	0.5533		B-C	0.9354
*Comparison of the percentage means of major compounds occurring in the dry season among the three individual plants*																				
*spathulenol*							*α-cadinol*							*τ-muurolol*						
ANOVA 1-factor				F (2,16) = 5.911		**0.012**	ANOVA 1-factor				F (2,12) = 7.055		**0.0094**	ANOVA 1-factor				F (2,16) = 0.06998		0.9327
	22EO (A)	6.004	0.621		A-B	0.0767		22EO (A)	7.334	0.3408		A-B	0.6579		22EO (A)	5.653	0.4532		A-B	0.9609
	80EO (B)	7.748	0.2299		A-C	0.506		80EO (B)	7.76	0.2616		A-C	**0.0458**		80EO (B)	5.79	0.194		A-C	0.9333
	530EO (C)	5.162	0.5943		B-C	**0.0104**		530EO (C)	6.03	0.4011		B-C	**0.0094**		530EO (C)	5.833	0.3482		B-C	0.9963
*Comparison of the percentage means of the major compounds occurring in the rainy and the dry seasons of the three individual plants*																				
*spathulenol*							*α-cadinol*							*τ-muurolol*						
t-tests				t = 0.1277 df = 39		0.8991	t-tests				t = 2.674 df = 39		**0.0109**	t-tests				t = 2.22 df = 46		**0.0314**
	Individuals RS	6.374	0.5252					Individuals RS	5.049	0.3869					Individuals RS	4.838	0.2323			
	Individuals DS	6.289	0.3799					Individuals DS	6.602	0.4359					Individuals DS	5.695	0.3173			

**Fig 3:**
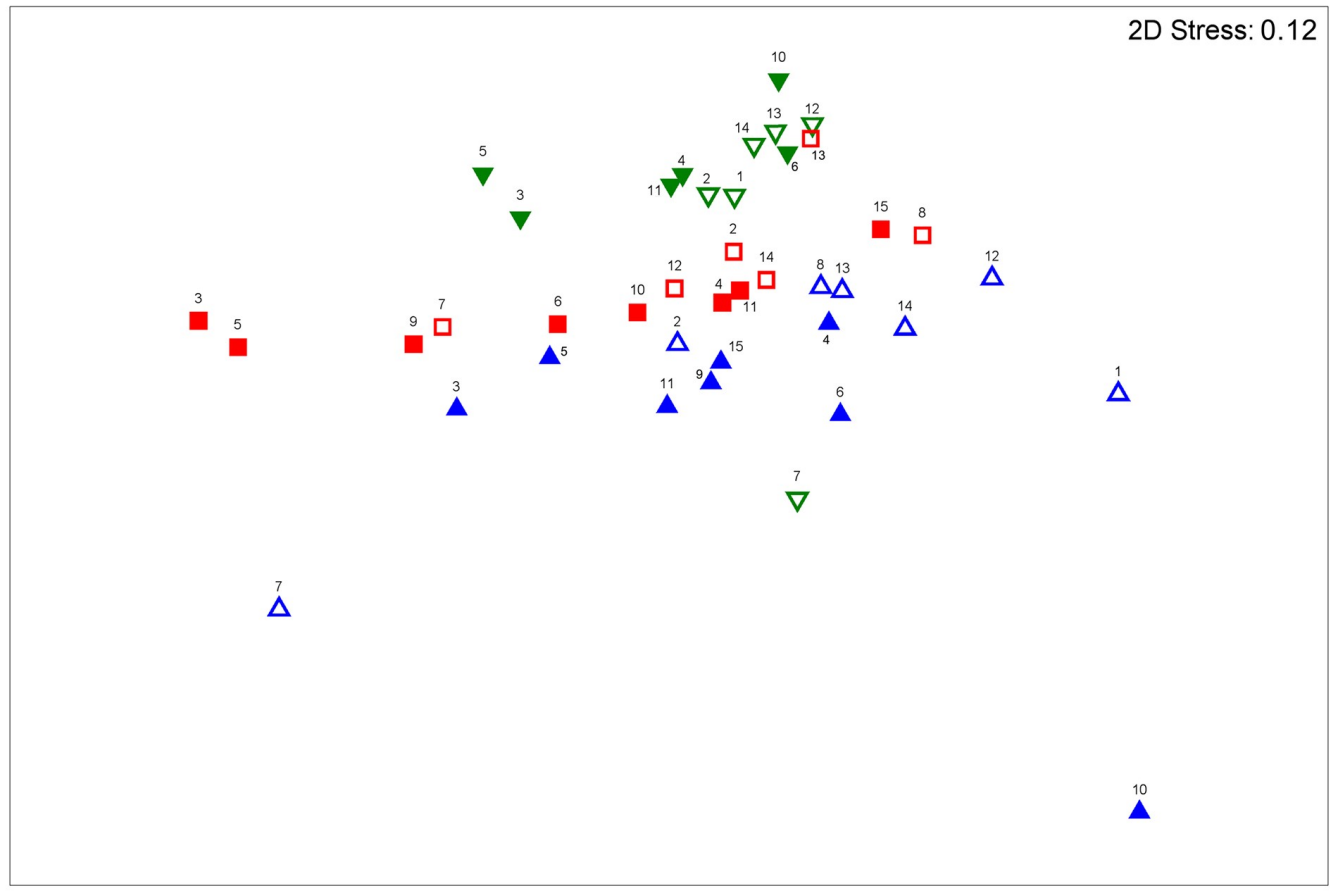
Ordination (NMDS) analysis of the 41 essential oils derived from three *Iryanthera polyneura* individual plants, using a Bray-Curtis Index (upward triangles = 22EO; downward-facing triangles = 80EO; squares = 530EO; shaded figures = rain season; open figures = dry season). The matrix used for the analysis was based on the percentages of the 59 terpenes found in the essential oils.

NMDS (*stress* = 0.12), in association with an ANOSIM, showed that the abundance and composition of terpenes in the samples varied significantly among the three *Iryanthera* individuals (R_Global_ = 0.284, *p* = 0.001) and also between DS and RS (R_DS x RS_ = 0.185; *p* = 0.038). Variation was determined according to calculations, because the values of the *R statistic* computed by ANOSIM vary from 0 to 1, with values closer to 0 representing indistinguishable groups of data. There were significant differences between abundance and the composition of terpenes between the two tested cases (all analyses were significant). However, the distinction between groups was not very evident. Considering only the three *Iryanthera* individuals tested, the groups that could be best distinguished (with respect to composition and abundance of terpenes) corresponded to individuals 80EO and 530EO (R_80EO vs. 530EO_ = 0.373, *p* = 0.001), followed by individuals 80EO and 22EO (R_80EO vs. 220EO_ = 0.342, *p* = 0.001). On the other hand, the group of samples of individuals 22EO and 530EO could scarcely by distinguished from each other (R_22EO vs. 530EO_ = 0.183, p = 0.008). These results are shown in Fig 3.

CA and CCA were performed to characterize oil composition according to the percentage of the major compounds spathulenol, τ-muurolol and α-cadinol. The utilization of such statistical tools makes it possible to observe spatial and temporal variations in the chemical composition of the oils considered [14, 23]. Further analyses allowed researchers to correlate geographical and environmental factors [24–26].

**Fig 4:**
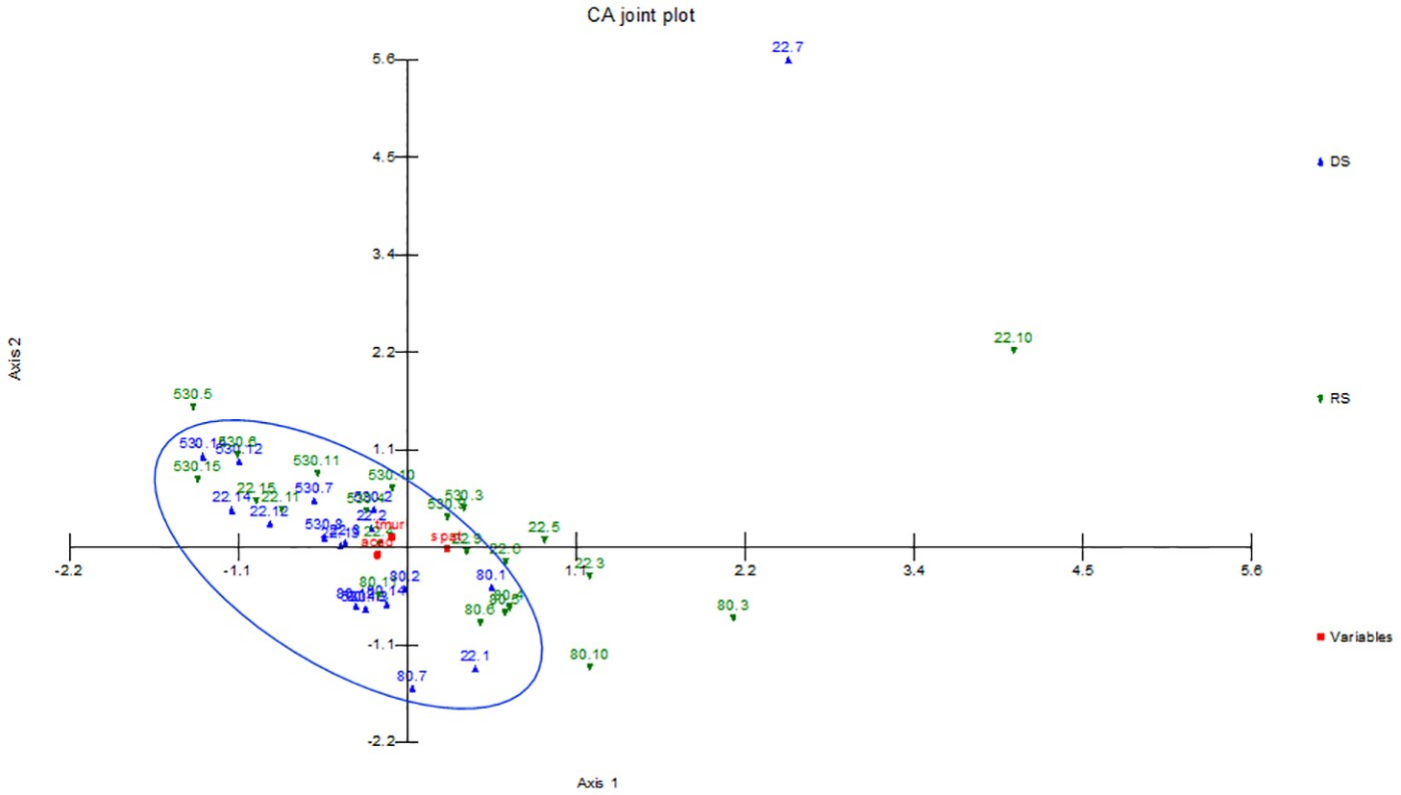
Correspondence analysis made with 41 essential oils (cases) obtained from the leaves of three *Iryanthera polyneura* individual plants (22EO, 80EO and 530EO). The major compounds spathulenol, τ-muurolol and α-cadinol are the variables. The numerical sequence (1 to 15) corresponds to different collection dates for each individual, fand increases according to chronology. Ellipses highlight the discriminated groups.

**Fig 5:**
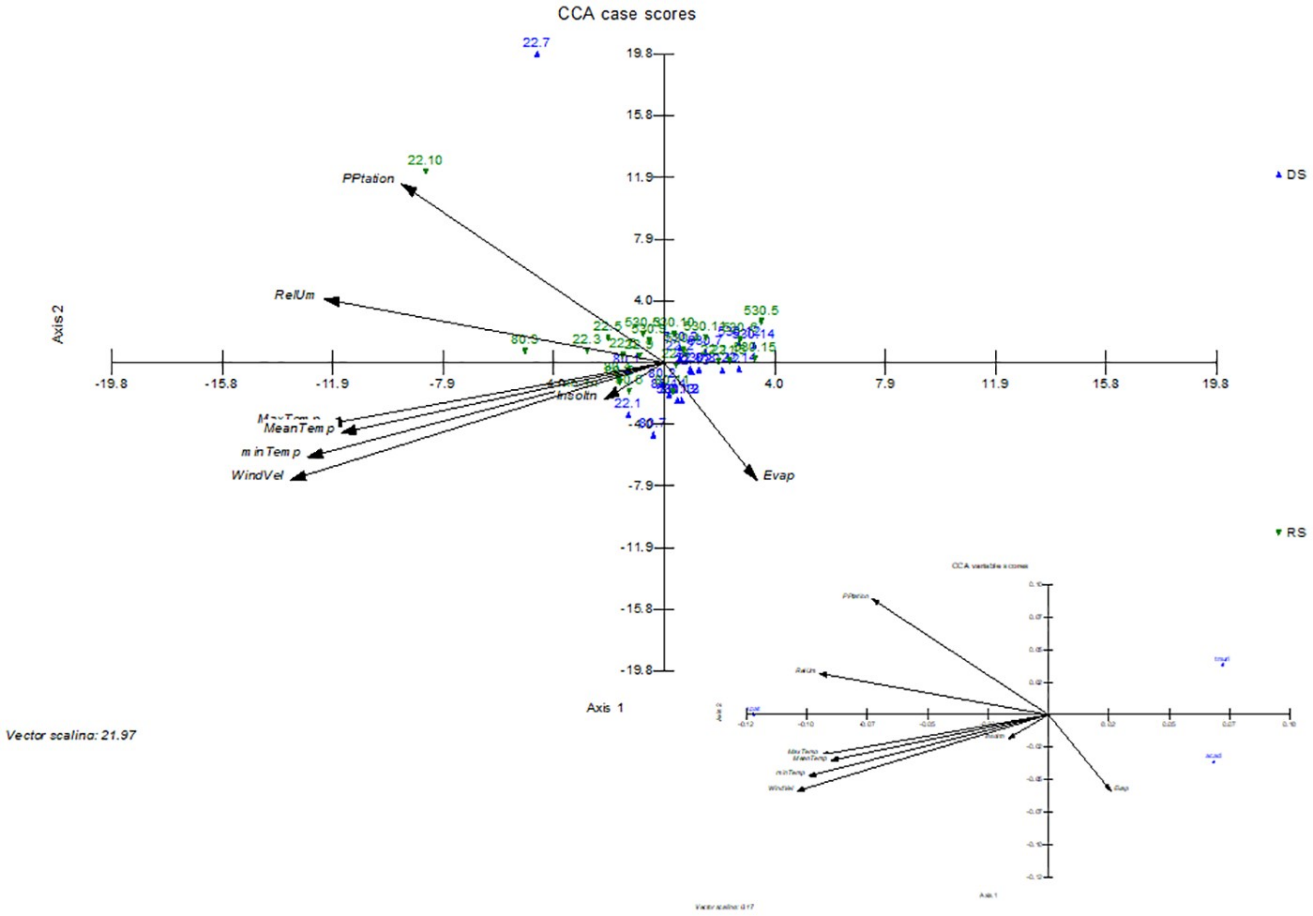
Canonical correspondence analysis made using 41 essential oils (cases) and three major compounds spathulenol, τ-muurolol and α-cadinol (variables). Oils were derived from the leaves of three *Iryanthera polyneura* individual plants (22EO, 80EO and 530EO). The numerical sequence (1 to 15) corresponds to different collection dates for each individual, following a chronological order.

Fig 4 shows a CA analysis made with the EOs from the three individuals (22EO, 80EO and 530EO) that has been based on the major compounds present on the oils. A cumulative percentage of 100% is shown on the second axis. The analysis revealed that the major compounds from the EOs varied based on whether they were obtained from leaves collected in the DS or the RS. The CCA analysis, shown in Fig 5, resulted in a cumulative constraint percentage of 100% on the second axis, and also showed a gradient, which was formed on the first axis with the samples of EOs obtained from the leaves collected in the DS and RS. Also, the CCA (Fig 5) showed that τ-muurolol and α-cadinol, which were more present in higher concentrations in the DS, according to the ANOVA first performed, are particularly influenced by the evaporation, rather than other climatic factors. Although the total amount of spathulenol does not change between the DS and RS (Table 4), the slight tendency of individual 22EO to have higher amounts of spathulenol during RS seemed to have been influenced by the precipitation and relative humidity (which are usually frequent during the RS), temperature and wind velocity.

### Conclusions

The findings presented have shown that there is a variation in the occurrence of the major compounds comprising EO from *I*. *polyneura* individual plants. The compounds spathulenol, τ-muurolol and α-cadinol varied among individuals and that the percentage variation was related to season and climatic changes.

## Supporting information

S1 Table Crude data from essential oil percentage. (XLSX)

S2 Table Biological data resulted from *in vitro* assays. (XLSX)

S3 Table Climate data from site of plant collection. (XLSX)
